# Cause or Effect? The Role of Prognostic Uncertainty in the Fear of Cancer Recurrence

**DOI:** 10.3389/fpsyg.2020.626038

**Published:** 2021-01-15

**Authors:** Paul K. J. Han, Caitlin Gutheil, Rebecca N. Hutchinson, Jason A. LaChance

**Affiliations:** ^1^Center for Outcomes Research and Evaluation, Maine Medical Center, Portland, ME, United States; ^2^Tufts University School of Medicine, Boston, MA, United States; ^3^Palliative Medicine Program, Maine Medical Center, Portland, ME, United States; ^4^Division of Gynecologic Oncology, Maine Medical Center, Portland, ME, United States

**Keywords:** uncertainty, fear of cancer recurrence (FCR), qualitative study, theoretical model, prognosis, ovarian cancer

## Abstract

**Background:**

Fear of cancer recurrence (FCR) is an important cause of suffering for cancer survivors, and both empirical evidence and theoretical models suggest that prognostic uncertainty plays a causal role in its development. However, the relationship between prognostic uncertainty and FCR is incompletely understood.

**Objective:**

To explore the relationship between prognostic uncertainty and FCR among patients with ovarian cancer (OC).

**Design:**

A qualitative study was conducted utilizing individual in-depth interviews with a convenience sample of patients with epithelial ovarian cancer who had completed first-line treatment with surgery and/or chemotherapy. Semi-structured interviews explored participants’ (1) understanding of their prognosis; (2) experiences, preferences, and attitudes regarding prognostic information; and (3) strategies for coping with prognostic uncertainty. Inductive qualitative analysis and line-by-line software-assisted coding of interview transcripts was conducted to identify key themes and generate theoretical insights on the relationship between prognostic uncertainty and FCR.

**Results:**

The study sample consisted of 21 participants, nearly all of whom reported experiencing significant FCR, which they traced to an awareness of the possibility of a bad outcome. Some participants valued and pursued prognostic information as a means of coping with this awareness, suggesting that prognostic uncertainty causes FCR. However, most participants acknowledged fundamental limits to both the certainty and value of prognostic information, and engaged in various strategies aimed not at reducing but constructing and maintaining prognostic uncertainty as a means of sustaining hope in the possibility of a good outcome. Participants’ comments suggested that prognostic uncertainty, fear, and hope are connected by complex, bi-directional causal pathways mediated by processes that allow patients to cope with, construct, and maintain their uncertainty. A provisional dual-process theoretical model was developed to capture these pathways.

**Conclusion:**

Among patients with OC, prognostic uncertainty is both a cause and an effect of FCR—a fear-inducing stimulus and a hope-sustaining response constructed and maintained through various strategies. More work is needed to elucidate the relationships between prognostic uncertainty, fear, and hope, to validate and refine our theoretical model, and to develop interventions to help patients with OC and other serious illnesses to achieve an optimal balance between these states.

## Introduction

Fear of cancer recurrence (FCR) is an important cause of suffering for cancer survivors and a topic of intense interest for clinicians and researchers. Broadly defined as “fear, worry, or concern relating to the possibility that cancer will come back or progress” (Lebel et al., 2016), FCR has been measured in different ways and found to be associated with various negative outcomes including psychological distress, diminished quality of life, and higher health care utilization (Lebel et al., 2013; Champagne et al., 2018; Mutsaers et al., 2020). Several theoretical models have been developed to explain the causes and effects of FCR, and the strategies patients use to cope with it (Lee-Jones et al., 1997; Fardell et al., 2016; Simonelli et al., 2016; Curran et al., 2017; Lebel et al., 2018). These models emphasize different factors and processes, but share a common view of prognostic uncertainty as a primary cause of FCR (Crist and Grunfeld, 2013; Simard et al., 2013; Hall et al., 2018; Tauber et al., 2019). Referencing Mishel’s influential Uncertainty in Illness Theory, several models construe uncertainty as a deficit state—specifically, an “inability to determine the meaning of illness-related events” (Mishel, 1988)—that provokes FCR both directly and indirectly (Fardell et al., 2016; Simonelli et al., 2016; Curran et al., 2017; Lebel et al., 2018).

This view of prognostic uncertainty as a primary cause of FCR is consistent with a vast body of research documenting the many aversive psychological effects of uncertainty (Afifi and Weiner, 2004; Han, 2013; Hillen et al., 2017b). At the same time, however, it reflects and reinforces a narrow conception of the nature, function, and beneficial effects of uncertainty. In treating uncertainty solely as a deficit state—a mere “inability” or absence of knowledge—it disregards how it also represents a form of knowledge in its own right: a metacognitive awareness of ignorance that serves the adaptive function of enabling individuals to cope with threats (Han et al., 2011). It overlooks potential beneficial effects of uncertainty, such as the maintenance of hope in the face of ignorance about the future (Babrow, 1992; Babrow and Kline, 2000; Brashers et al., 2000; Brashers, 2001). This benefit may explain why patients with serious life-limiting illness strive not only to decrease prognostic uncertainty, but to increase and maintain it in various ways—including actively avoiding prognostic information and embracing uncertainty in prognostic estimates (Fallowfield et al., 1995; Butow et al., 1997, 2002; Kutner et al., 1999; Hagerty et al., 2004, 2005; Kirk et al., 2004; Helft, 2005; Elkin et al., 2007; Hancock et al., 2007; Robinson et al., 2008; Innes and Payne, 2009; Bilcke et al., 2011; Rayson, 2013; Han, 2016). These responses suggest the need to move beyond construing prognostic uncertainty solely as a cause of FCR and other psychological states, to adopt a broader, bi-directional view that accounts for the alternative role of prognostic uncertainty as an *effect* of FCR—a consequence of patients’ efforts to cope with their fear.

The overarching aim of the current study was to explore this alternative role and the potential bi-directional causal pathways that link prognostic uncertainty and FCR among patients with ovarian cancer (OC). The most lethal gynecologic malignancy and fifth leading cause of cancer deaths among United States women, OC has a high risk of recurrence and progression and OC survivors have a correspondingly high degree of FCR (Wenzel et al., 2002; Shinn et al., 2009; Roland et al., 2013; Ozga et al., 2015; Kyriacou et al., 2017; Galica et al., 2020). The experiences of OC survivors may thus yield valuable insights on the causes and effects of FCR, and the specific aim of the current study was to explore these experiences to better understand how prognostic uncertainty might represent not only an aversive cause but an adaptive effect of FCR. Appropriate to this exploratory aim, the study employed in-depth, semi-structured qualitative interviews with individual OC survivors, which allowed survivors themselves to describe their lived experiences in detail, and to account for the relationships between prognostic uncertainty and FCR in their own words. The study thus allowed us to generate testable hypotheses about these relationships and to develop a tentative, provisional theoretical model to guide future research on the relationship between prognostic uncertainty and FCR among cancer survivors.

## Materials and Methods

### Study Design, Participants, and Recruitment

The study utilized individual qualitative interviews with a convenience sample of patients with epithelial ovarian or fallopian tube cancer, recruited from the Gynecologic Oncology Service of a large urban 637-bed teaching hospital. Eligible participants consisted of patients with Stage I–IV disease, determined by surgical or clinical staging after biopsy-confirmed diagnosis, who had completed first-line treatment with surgery and/or chemotherapy. In order to minimize potential psychological harms that might be caused by asking patients to discuss prognosis-related topics including the prospect of their own death, patients were excluded if they reported significant levels of cancer-specific emotional distress on routine screening using the National Comprehensive Cancer Network (NCCN) Distress Thermometer (Holland et al., 2001) (scores ≥ 3). Eligible and interested patients were identified by practice staff during regularly scheduled office visits and recruited by the research team, who informed participants of the study’s voluntary and exploratory nature and its overall focus on the topic of prognosis. Participants were provided a $25 (USD) incentive. We aimed for a minimum sample size of 20 based on available study resources and our prior qualitative research exploring similar themes with cancer survivors (Han et al., 2013; Hillen et al., 2017a). The study was approved by the Maine Medical Center Institutional Review Board.

### Data Collection and Interview Content

From July 2018 to November 2019, individual in-person interviews, lasting approximately 45–60 min each, were conducted by trained qualitative researchers (CG, HM, PH) with no professional or personal relationships with participants. Interviews were semi-structured and followed a moderator guide developed by our multi-disciplinary research team and consisting of open-ended questions and close-ended probes designed to elicit participants’ (1) prognostic understanding; (2) experiences, preferences, and attitudes regarding prognostic information; and (3) strategies for coping with prognostic uncertainty (Appendix). During the course of the study, minor revisions were made in the interview guide to clarify and further explore emergent themes. All interviews were audio-recorded with prior consent of participants, and transcribed verbatim by a professional transcription service.

### Data Analysis

In-depth qualitative analysis and line-by-line software-assisted coding of anonymized interview transcripts was conducted using the qualitative analysis software program MaxQDA^TM^. The analysis utilized an inductive, constant comparative approach aimed at minimizing preconceptions, identifying key themes and relationships between them, and generating new theoretical understandings (Glaser, 1965; Strauss and Corbin, 1998). Four investigators—a palliative medicine physician and behavioral researcher with expertise in medical uncertainty (PH), an experienced qualitative health researcher (CG), a palliative medicine physician and health services researcher (RH), and a medical sociologist (AF)—first developed a working codebook by reading eight transcripts, inductively identifying themes in participants’ verbatim statements (open coding), and then categorizing emergent themes according to their content (axial coding) (Strauss and Corbin, 1998; Ryan and Bernard, 2003; Corbin and Strauss, 2014). The investigators met after coding each transcript to compare coding decisions, resolve areas of disagreement, and refine the codebook. The remaining 13 transcripts were double-coded by pairs of researchers (PH and CG, or PH and RH), who met regularly to compare new data, concepts, and themes, to resolve further disagreements, and to refine the codebook. One researcher (PH) conducted further analysis of identified themes and developed an integrative conceptual model with feedback from the research team.

## Results

The study sample (Table 1) consisted of 21 participants, most of whom reported experiencing prognostic uncertainty and FCR at some point in their illness. Their descriptions of the relationship between these phenomena suggested two different views: (1) prognostic uncertainty as a cause of FCR, and (2) prognostic uncertainty as an effect of FCR. Supporting the former view, participants described managing FCR by pursuing prognostic certainty. Supporting the latter view, participants described managing FCR by pursuing prognostic uncertainty, and further identified various strategies that they used to construct and maintain this uncertainty. These views, furthermore, were not mutually exclusive; some participants endorsed both at once.

**TABLE 1:** Participant characteristics.

	***N***	**%**
**Age**		
40–50	4	19.0
51–60	3	14.3
61–70	9	42.9
71–80	5	23.8
>80	0	0
**Race**		
White/Caucasian	21	100
Other	0	0
**Cancer type**		
Ovarian	16	76.2
Fallopian tube	5	23.8
**Cancer stage**		
I	3	14.3
II	1	4.8
III	14	66.7
IV	3	14.3
**Months since diagnosis**		
≤ 12	8	38.1
13–24	4	19.0
25–36	4	19.0
37–48	1	4.8
49–60	2	9.5
>60	2	9.5

### Prognostic Uncertainty as a Cause of FCR

Consistent with prevailing theories of FCR, study participants described prognostic uncertainty as a primary cause of their fear, which they managed by pursuing prognostic certainty.

#### The Fear of Possibility

Study participants’ accounts suggested that prognostic uncertainty was associated with higher FCR. Participants reported experiencing the greatest uncertainty and fear at the time of their initial cancer diagnosis, and during follow-up visits and diagnostic testing (bloodwork and radiologic imaging studies) for cancer surveillance. Participants’ comments further suggested that FCR originated not only from uncertainty *per se*, but from a negative bias in its perception and interpretation: a selective, pessimistic focus on worst-case possibilities, which clinical psychologists have termed a “catastrophizing” response to uncertainty (Robichaud, 2013). This bias was manifest in several study participants’ tendency to equate prognostic uncertainty with worst-case outcomes including cancer recurrence, progression, and death (Table 2). One participant (Patient 15), for example, traced the fear she felt at the time of her routine follow-up visits both to the inability to know whether or not her illness “was going to end badly,” and to an irresistible urge to imagine the worst: “every time I come here I’m panicked, just because it is scary to think that I could come back at any time and have a re-occurrence.” Other participants exhibited the same pessimistic bias in describing their cancer as a “hiding,” “sneaky” threat lurking “somewhere else, just waiting” to return and spread.

**TABLE 2:** Prognostic uncertainty as a cause of fear of cancer recurrence.

**Themes**	**Illustrative quotes**	**Patient**
*The fear of possibility*	I mean it’s cancer. And cancer is sneaky. For me, you hear of cases that are positive but you also hear of cases that are negative where it wasn’t supposed to come back but it did. So I think the percentages would have to be way higher, like 99%, you know. And it’s just not that high. So 80—I forget what it is, 80 to 90% is great, but it’s not 100% chance of it not coming back.	17
	But I have to say, just that initial diagnosis when they say, “Oh we think you possibly have ovarian cancer or you do have ovarian cancer,” it’s almost like a PTSD thing. So emotional, crying, fear. That fear is with me all the time. It’s always like, any little thing I get now, I think, has that cancer spread? Do I have cancer somewhere else now? Because cancer always comes back … But just the diagnosis, knowing that they found cancer in an ovary, it’s in my head. I had cancer. And that fear is in me that it’s somewhere else, just waiting … They say, it was tiny, we got it, it was nowhere else. But in my head it was nowhere else that you looked, and what if there’s one tiny little thing and it’s somewhere just waiting years down the road. Because you hear people beat cancer and then 15 years down the road it’s back. So I have that and I get really emotional. … I’m like, “I’m a nervous wreck, I’m crying because I’m scared to death.”	20
	Well, now being in cancer—knowing what I know about it, you’re not really in remission, it’s just hiding really. It doesn’t really ever go away … if you read the Internet, high-grade serous carcinoma almost always comes back. I’m not sure I’ve found anybody that it hasn’t. It doesn’t mean it’s going to kill you. It just means it flares again. You know, your CA 125 starts going up. So they start hitting you with more chemo. Or it goes to an organ. Because now everything I have now is vital. They took out everything I didn’t need. So if it goes—like it likes to go to the lungs. It likes to go to the spleen, the liver, sometimes the bowels and the bladder, sometimes the brain, yay!	21
*Restricting possibility: the need for certainty*	I don’t believe the number absolutely. But it gives me a time bracket. So, I shan’t be worried about things that are going on that I can’t explain, because they’re either old age or cancer. But after this point, if something different starts to happen, I will make note of it. Up until that point, I could probably drop dead in 4 years and not worry about it. But it does give me a sense of time. For me that’s good.	12
	I was trying to get reassurance on—I was really looking for stats, like this percentage of people survive … Again, in my case just looking for reassurance like okay, for a person between the ages of 40 and 50 who have BRCA and get it caught in Stage II, what are the numbers? They’re not always available. But for me it was just kind of trying to reassure myself that in general ovarian cancer is a pretty—is one of the harder ones to treat successfully in the long run. So I’m just looking for something like when she says this is curable, this is beatable, you’re in a good situation, I just want to see the numbers that back that up I guess.	17
	What’s my chances of dying? That’s what everybody wants to know when they get cancer. But I mean, I do think it’s good to know. Most of that stuff I’ve learned, like I said, from the Internet. But maybe it would be helpful to know, what is the likelihood that it’s going to come back? When it will come back? So I’ll know when to—sometimes concrete knowledge is good … You want to know. You don’t want to live and la-la land … You want to be making plans. Should I be working at this point or should I be out making memories with my family? So that’s what I mean. I think it kind of helps with the seriousness. Ovarian cancer is not the worst, but it’s not the best. You’ve got a 50/50. Okay, so what makes up that 50/50? Tell me about the people that are on the bad side of 50.	21
*Unrestrictable possibility*	… let’s say I’m in the 74%, who knows which side I’m on. The 74 or the 26 side. Who knows? Who knows?	3
	We think that probably based on that and study one, 40 days, you know, all this stuff, probably you’re gonna be fine. If he says 60% or 80% does that really mean anything to me? No. That’s bullshit. It’s word filler. and so I just want to hear the real stuff. And if it’s not real, just keep it to yourself.	9
	That was sufficient for me. I would have, me, if he would have said, you have 0%, I would have stuck to that. If he said you have 0 to 0.5%, that 0.5 would drive me crazy forever. I would focus. I’d still have a 0.5. This past May I was having a lot of dizziness and stuff. So anyway, long story short, I ended up in the Emergency Room and they were doing heart monitor, then they look at my history and they’re like, hmm, “Well we better do an MRI on your brain and a CT scan, because you’ve had Ovarian Cancer.” Well hello. That’s what I mean by it’s always there. Always there.	20

#### Restricting Possibility: The Pursuit of Certainty

Further supporting the view of prognostic uncertainty as a primary cause of FCR, participants reported managing their fear by pursuing prognostic certainty. This pursuit served the critical function of restricting future possibilities—that is, narrowing the range of potential outcomes—which made them more tolerable (Table 2). One participant (Patient 12) argued that reducing her prognosis to a finite “time bracket” gave her a “sense of time” that lessened her worry. Other participants (Patients 17, 21) similarly reported that by limiting the range of possible outcomes, prognostic information imposed a concrete order that enabled them to set priorities in their lives—a function they perceived as valuable regardless of how unfavorable their prognosis might have been.

#### Unrestrictable Possibility

At the same time, study participants acknowledged fundamental limits in the extent to which prognostic possibilities could be restricted. Participants argued that medical experts “don’t really know” the prognosis of individuals (Patient 2), that statistics “are not about people” because they simply “mix together” individual lives (Patient 7), and that even precise prognostic estimates do not answer the question of their own fate (Table 2). Participants thus recognized that the range of their potential outcomes could never be narrowed sufficiently to include knowledge of their own personal fate; prognostic certainty was unachievable. This recognition was a source of distress for many participants; Patient 20, for example, reported that even a small “0.5%” probability of a bad outcome was enough to “drive me crazy forever.” Such responses lend further support to the view of prognostic uncertainty as a primary cause of FCR.

### Prognostic Uncertainty as an Effect of FCR

Yet study participants’ accounts of their experiences suggested that prognostic uncertainty was not merely a primary cause but also a secondary *effect* of FCR: an adaptive response as well as an aversive stimulus, a source of hope as well as fear (Table 3).

**TABLE 3:** Prognostic uncertainty as an effect of fear of cancer recurrence.

**Themes**	**Illustrative quotes**	**Patient**
*The hope of possibility*	So I am fighting cancer. Even though nothing shows on a scan right now, it doesn’t mean there’s not still cancer. And I’m not in denial about that, you know. I’m fighting it. You know everyone will say that; I don’t know how many times I’ve heard, “Oh I’m so sorry.” I say, “Why? I’m going to beat it.” That’s what I say every time. I say, “I’m beating it now.”	4
	Well I mean also I feel because I’m on this new treatment, you know because, I mean my provider has straight up said, I have no idea; you’re in the wilderness and we have no statistics; you’re the, you’re one of the like, you’ll be a, you know you’ll be a statistic. Like I’m part of the like, “Does this work, or does it stop working after 5 years, or does it give me another kind of cancer, or?” I mean all of those are—like it’s all possibilities. So there’s something really liberating in that in a way … Yeah, you never know, you never know. Like you could just—like I mean yes, the natural history of this disease is most people are dead within X number of years; you know you have some recurrences, they get closer together, they don’t, they get resistant to treatment and then you die a terrible death, right? So yeah, so not knowing, like just being like, just having the, maybe this will work for a long time, like creates a little more hopefulness.	7
	But it was never, for me personally, it was never a question that I needed to ask. I already knew the answer and how I know, I don’t know. But it was always a matter of, “Okay, I’ve got this. Let’s get on with the treatment. And of course I’m going to survive” … And then he gave us that information. But it was like 41/2 years survival rate for women with III-C or greater, IV. And I looked at him and I said, “So half of the people don’t make it to 41/2 years. And I said, “And half of the people live way beyond that.” I said, “I’m definitely in the second category. Definitely.” I said, “There’s no question about that at all.” And it was, it’s not, and even when I was at the bottom looking up at the bottom of the barrel, it was never a point of I’m not going to get through this. It’s like, “Yeah, this is hard, and this doesn’t feel good. And this is the pits, but tomorrow is better. Tomorrow you will feel better. Tomorrow you will be able to do a little bit more than you did today. Don’t worry about today. Tomorrow is going to be better.	14
*Expanding possibilities: the need for uncertainty*	… the first time I met with Dr. _______ I said, “I don’t want to know what stage cancer it is because if I don’t know, it can be anything.” Do you know what I mean? So I didn’t want to know that because if I knew a number, that’s going to, that’s going to be a scary thing. And I didn’t, I didn’t want to know a number. I knew it was serious; hell, I knew it was serious. I’ve got this big tumor; they’ve all told me it’s an adeno something or other; it’s aggressive … I know that, I know that that means it’s stage 4. But as long as they’re not telling me that, I haven’t heard it.	2
	So maybe that’s why I didn’t want to know originally. I didn’t want any, to know like, “Well this could be really bad,” because I’d be thinking, “I’m not going to survive, you know” … I didn’t ask questions that I didn’t think—I mean, “Am I gonna die?” “What are my chances?” I think those are questions that a doctor can’t answer with any more certainty than I can. And rather than hear him guess or not, and distort my confidence in him, only ask a question if you really want the answer.	8
	I don’t want to think about it too much and if there’s hope then I’m good with that. I didn’t really want, and quite honestly I kind of knew that it probably wasn’t gonna give me good numbers. I was at like Stage 3, so I didn’t anticipate—I was just kind of grasping, kind of hoping for a positive to hang onto … I did get paperwork and stuff when I first, one of my first times here when they were explaining things to me, and it had statistics and stuff. And I looked at ‘em once, and I couldn’t look at them again because they were not helpful to me. I only wanted to hear words of encouragement and hope. And those statistics did not give me any of that … I didn’t really want the numbers. I just wanted at that time to hear from him, “Oh, you’ve got this,” or whatever. And it was wishful thinking on my part to even to ask. So in response to your question, no I wasn’t really looking for numbers necessarily … If it was something that was going to give me hope and like, I can do this sort of thing, then I probably would have wanted them, but if it was anything negative I wouldn’t have wanted it.	15
*Adaptive effects of uncertainty*	Well to finalize something that—you always have that on your mind. You’re counting the days. Like say if I was told I had 3 months, you’d be living every day just counting the days away. That type of thing. And not trying to focus on getting better, or you know, prolong it or whatever.	13
	So, I’m good with that. I tend not to borrow trouble. It’s really, what do I have to do now? Where do I have to be? What do I have to do? What do I have to take? And just tell me when and where to be there and I’ll do my part … Here’s where I am. I’m doing everything that I can do. Dr. ____ is doing everything that he can do. There is nothing else to be done. So I’m not doing any more.	9
	I guess the biggest thing is I am such a worrier, and I would think about it too much and those—for me, not knowing everything helped because I knew what I knew and what I was working on and that’s all I could control, and beyond that I had to trust the care I was getting.	15

#### The Hope of Possibility

Study participants’ accounts suggested that prognostic uncertainty was associated not only with higher but lower FCR. Participants’ comments further suggested that this association may have originated from a positive bias in the perception and interpretation of uncertainty: a selective, optimistic focus on best-case possibilities, which might be termed “optimizing”—in contrast to the “catastrophizing” that resulted in the fear of possibility. This opposing focus made prognostic uncertainty a source of hope as well as fear. One participant (Patient 5) viewed even the smallest possibility of a best-case outcome as a reason for hope: “even if there was a 1% chance, I was like it didn’t matter,” she asserted, adding that “We don’t know what they can do.” In a similar vein, Patient 4 focused on the possibility of “beating” her cancer, in deliberate defiance of the high likelihood that her cancer would recur. Patient 7 acknowledged the “liberating” nature of the fact that all future outcomes—both bad and good—ultimately represent mere possibilities, and affirmed how the awareness of this fact “creates a little more hopefulness” (Table 3). For all of these participants, in other words, prognostic uncertainty signified the possibility of not only realizing a dreaded outcome—cancer recurrence, progression, death—but averting these outcomes. Uncertainty was thus a source of not only fear but hope.

#### Expanding Possibility: The Pursuit of Uncertainty

Corroborating the alternative view of prognostic uncertainty as a secondary effect of FCR rather than a primary cause, study participants reported coping with their fear by increasing rather than decreasing their uncertainty. Prognostic uncertainty was a key goal for many participants, who described conscious efforts to maintain ignorance about their future as a way of expanding the range of prognostic possibilities to include desirable as well as undesirable outcomes (Table 3). Patient 2 put it pointedly: “I don’t want to know what stage cancer it is because if I don’t know, it can be anything.” Patient 15 acknowledged her poor prognosis but reported actively foregoing more precise prognostic information—intentionally maintaining prognostic ignorance and uncertainty. For this and other patients, uncertainty kept prognostic possibilities open, and thus served the vital function of preserving hope in a better future.

#### Adaptive Effects of Uncertainty

Related to—yet independent of—its hope-preserving function, furthermore, prognostic uncertainty had other important, psychologically adaptive effects (Table 3). One was to prevent patients from resigning from further efforts to fight their disease; Patient 13 viewed such a response as a danger of prognostic certainty and a rationale for preserving prognostic uncertainty. Another adaptive effect of uncertainty, acknowledged by multiple study participants, was to help them focus attention on issues within their control and to disengage from issues that were not. One participant (Patient 9) viewed maintaining prognostic uncertainty as part of a general strategy “not to borrow trouble”—to let go and simply attend to the pragmatic task of “doing what I can do.” Other participants affirmed that prognostic uncertainty had the added benefit of allowing them to cease thinking and worrying about their future outcomes, as Patient 15 put it—and to “trust the care I was getting.”

### Constructing Prognostic Uncertainty

Further supporting the conception of uncertainty as an effect rather than a cause of FCR, study participants not only pursued prognostic uncertainty by foregoing prognostic information, but actively constructed it by engaging in two strategies: (1) highlighting the indeterminacy of their personal prognosis, and (2) highlighting ambiguity in prognostic information (Table 4). These strategies reinforced participants’ prognostic uncertainty, which further enabled them to maintain hope in a favorable outcome.

**TABLE 4:** Constructing prognostic uncertainty.

**Themes**	**Illustrative quotes**	**Patient**
*Highlighting indeterminacy*	I am a fighter. I am my mother’s daughter and she’s a fighter and we don’t ever follow the rule book, so if the rule books say 20%, then screw that. We’re going to change the rule books. And you know, and we’re all like that in my, my mom’s side anyway and I just, no I just never thought I was going to go down like that, and so I just was determined not to … you know when they told me 20%, I said, okay so somebody has to be the one that skews the odds. I said, and why can’t I be that person? So that’s just how I viewed it from the day he told me so. I didn’t let the 20% affect me that way … You know that’s funny because I think that certain people obviously are different. Everyone is so individual … But like I said, I’m not the average Joe. But I think that, so maybe I feel that maybe some doctors, hopefully they gauge out; I mean they only know you as a chart.	4
	So you’ve got to find yourself a different Cinderella, this shoe doesn’t fit me. I’m not anywhere close to calling it quits … The objective point of view is, you’ve got all of this information coming in. Yeah, it’s legit for him to say that. But then you have the part that he can’t see, which is the subjective part, which says yeah, but you know what, you know better than that … the subjective part that they can’t see and they don’t know about or they can’t hear, is the greater part of that equation. And that part of the equation is the part that you hang onto. And not just hang onto, but is the real part, is the missing bit of information to go into this equation to turn that answer around.	14
	There is some backing for statistics. Yeah, I’m sure there’s some research behind it and stuff. I know, well, I don’t know this but this is what I think. I know often times people who have ovarian cancer tend to be older, I think. And maybe the statistics have to do with them a little bit more … But I was just thinking that maybe the statistics have to do more with them than maybe me … I remember asking Dr. ___, “So am I going to be okay?” And he couldn’t exactly answer that question, obviously. But he did say, “Every patient is different.” He said, “I have patients that don’t do well.” And then he said, “I have patients that do amazing.” And so he said, “I can’t answer that question” … I think he was just honestly saying, “I don’t know exactly what’s gonna happen, how you’re going to react to the treatment.”	15
*Highlighting ambiguity*	I wanted to dissect the number right away. Okay, you say let’s say 40%, and then I go in and I read ovarian III on the internet and it says women over 65 have a poorer prognosis than those who are under 65. Okay, I’m 72. So how many of that 40% are under 65 and how many of them are over 65? If you’re in the 70’s, are you telling me the 40% has all of the women who are 28 years old all the way up to 90, and I’m probably actually more like I’ve only got a 28%, or are you telling, you know. I wanted to dig into it but I also knew and they said they can’t, they, we can’t tell you; that research hasn’t been done.	3
	And I had another friend who had had cancer. And they both said to me, don’t look at the research numbers in terms of healing or how long you’re going to live because that research was done 5 years ago at least and so many innovations have come from that since then.	5
	Yeah, because what we know today, what I knew or what they knew in September of ‘16 is different than what we know of what this is like in April of’19, things have happened. More drugs have come out. And that was one thing that they talked about saying, “Well these two drugs aren’t for you anymore but we have this one and this one and this one. We’ve got an arsenal. We’ve got a toolbox full of drugs that we can give you” … Now it’s open-ended. Depending on how soon you get treated, how soon you get diagnosed, see how much further information we have to give you to say, “Yeah this is a devastating diagnosis to you, but because of all the research we’ve done and because of all the studies that we’ve done, we know that at your stage, this is how long, a productive life you have. And that’s hopeful.	14

#### Highlighting Indeterminacy

The most commonly reported way in which study participants constructed uncertainty was by highlighting the indeterminacy of their prognosis. Nearly all participants expressed a belief in the uniqueness of individual patients, and in the inability of statistics to account for all possible determinants of their own, personal prognosis—e.g., age, medical history, family history, positive attitude, and lifestyle. These beliefs affirm long-recognized epistemological problems that limit the precision and value of all probability estimates for individuals, and engender what philosophers of statistics have called aleatory uncertainty (Hacking, 1975; Gillies, 2000). Study participants, however, embraced and highlighted this uncertainty because it allowed them to believe that prognostic estimates did not apply to them, and that their personal prognosis was thus alterable. Folkman (2010) referred to this cognitive reappraisal process as “personalizing the odds.” As Patient 4 affirmed, “I think that certain people obviously are different. Everyone is so individual”; she went on to assert that she was “not the average Joe” and would “change the rule books” in fighting her cancer (Table 4). Another patient stated that she purposefully avoided seeking prognostic information because “Whatever it [prognostic estimate] says, I want to be the outlier” (Patient 5). The effort to highlight prognostic indeterminacy to preserve the possibility of beating the odds, furthermore, appeared to involve physicians as well as patients. Participants recounted how their physicians used language supporting the personal inapplicability of prognostic information—e.g., “Every patient is different” (Patient 15), and “your situation is a little different” (Patient 17). Patient 21 stated that her physician refrained from providing any prognostic estimate precisely for this reason: “And she’s never—she still hasn’t given me survivability odds. She just says that I am my own case. She says, ‘You know it’s really difficult to give that kind of definite knowledge because everybody is so individual.”’ These accounts suggest that physicians played an important role in co-constructing prognostic indeterminacy with patients as a means of maintaining hope in beating the odds.

#### Highlighting Ambiguity

Another important way in which study participants constructed prognostic uncertainty was by highlighting limitations in the reliability, credibility, or adequacy of prognostic information—features of information that produce what decision theorists have termed “ambiguity” (Ellsberg, 1961; Camerer and Weber, 1992). Ambiguity in prognostic information arises from missing or conflicting risk evidence or methodological limitations that produce imprecise, conflicting, or changing estimates, leading to what philosophers of statistics have called epistemic uncertainty. Study participants highlighted the ambiguity of prognostic information in several ways, including acknowledging the imprecision or unreliability of prognostic estimates for particular patient subgroups, due to shortcomings in empirical evidence—e.g., “research hasn’t been done” (Patient 3) (Table 4). Other participants noted how prognostic knowledge is unstable and “changing at every moment” (Patient 21) due to ongoing scientific advances. Highlighting these ambiguities of prognostic information reinforced participants’ uncertainty, further broadening the range of their possible futures to include favorable outcomes.

### Maintaining Prognostic Uncertainty

Study participants described how they not only construct uncertainty by acknowledging key limitations of prognostic information, but actively maintain it by engaging in two other general strategies—relinquishing knowledge and embracing ignorance—which enabled them to both sustain hope and better tolerate their uncertainty.

#### Relinquishing Knowledge

A primary strategy that participants used to maintain prognostic uncertainty consisted of relinquishing prognostic knowledge and its pursuit. Several participants viewed their prognosis as a moot question given that only one course of action—to pursue treatment—offered any hope of controlling their cancer; prognostic knowledge thus served no practical purpose. In this vein, Patient 2 argued that simply knowing she had OC was enough, and that her priority was simply “to concentrate on what we can do,” while Patient 4 disavowed prognostic knowledge because it would not change her “plan of care” (Table 5). Other participants added that prognostic knowledge was not only practically but existentially irrelevant. Patient 18 affirmed a need to accept whatever future outcomes might lie ahead, and to simply deal with her illness one step at a time. The practical and existential irrelevance of prognostic information led these participants to relinquish prognostic knowledge and its pursuit—especially as time went on and their illnesses progressed—which had the ultimate effect of sustaining their uncertainty and making it more tolerable.

**TABLE 5:** Maintaining prognostic uncertainty.

**Themes**	**Illustrative quotes**	**Patient**
*Relinquishing knowledge*	Believe me; hearing I’ve got ovarian cancer; that’s enough. That’s going to make my head spin. I don’t need the extra 5 min on the whirligig you know. It’s like, I’m getting enough information with that piece of information. And this, hmm, how do I word this? I think sometimes we do think; like we put things in numbers, in categories where really it doesn’t need to be … so people with stage I have a greater chance of recovery, and people from stage 4 have a less chance of recovery. Does that help? Does knowing that really help? … Seriously, I don’t think knowing those numbers helps anybody; I really don’t think it helps because like I said, it, they’re just numbers. And like Dr. ________ said, it’s a number; you’re one in a thousand and it’s what it is you know … we’ve got to concentrate on what we can do.	2
	I don’t know that doctors may not tell their patients how grim it is because, well like he told me; no matter what, if it was in my bones or if it was, then the course of treatment was going to be the same. I was still getting these six cycles of chemo, this chemo, no matter what the course was. Whether it was in my bones or whether it was everywhere or if it was just here … So in my mind, I’m thinking that he didn’t give me all of that total information because it wasn’t changing my plan of care …. It isn’t going to make a difference. Because the treatment’s the treatment. And what you have at the end is what you have at the end.	4
	No. I’m not worried about it because if it’s gonna happen, it’s gonna happen. I’m strictly like—she said to me today, “I’d like to do a CT scan, I think it’s come back.” Okay, let’s do the CT scan. And he said, we’ll start treatment again.” “Okay, let’s do treatment again.” I’ve just accepted it. Is that understandable? I’ve accepted that I have cancer. And I’ve just gotta do it.	18
*Embracing ignorance*	So I think that’s my, the way I am … it’s like we don’t need to know. We’re suited to take it a day at a time and yet, I mean, it’s okay to plan somewhat; I’m not saying that. Like okay, if suddenly we couldn’t stay with our family, then that’s okay; we kind of know, we’re free, we can do this. Well there are other options you know. That’s been our life … Yeah, we’re cut out for that I guess but it’s very freeing.	1
	For me, it wasn’t helpful because I didn’t want any limit. I watched it with my mother. I watched what limits do to people across every, any level. If you tell someone who’s a quadriplegic that they can’t ski and then you go up and you see them on the mountain, the kids that weren’t told that they couldn’t. And I just think that for me, I don’t want to know; I didn’t want to know what my chances were or what my odds were … I’ve watched it with friends who get diagnosed with something and they, that’s what they; you know I only have 5 years, I only have, the doctor said you know I have 3–5 years and that’s all they think about and I thought, “I have today…”	8
	I mean again, it’s because I think it has to do with the, like providers holding some amount of the spaciousness of the unknown. Like here’s what we can do and here’s what seems to work but they don’t know and I think that’s something in medicine overall. Like it’s about fixing; not about health … I mean you know you can’t push it but if you hold the space, if you hold the space for it, there’s an invitation also to, for us as patients to like drop a little under. I mean some people can’t.	7

#### Embracing Ignorance

Yet study participants reported maintaining prognostic uncertainty by engaging in not only the negative act of relinquishing knowledge about their prognosis, but the positive act of embracing ignorance. Apart from the basic desire to maintain prognostic ignorance in order to expand the range of future possibilities to include hopeful outcomes (Table 3), participants also described a positive affinity for ignorance, which served other fundamental life goals. Patient 1 reported that embracing ignorance about her future provided a sense of freedom and openness that allowed her to “take it a day at a time” (Table 5). Patient 8 embraced ignorance about her prognosis because she “didn’t want any limit” to what was possible; she believed that acknowledging a poor prognosis posed the risk of turning into a self-fulfilling prophecy. Patient 7 evocatively described this same orientation toward ignorance as a capacity for “holding some amount of the spaciousness of the unknown”—for affirming prognostic ignorance and preventing it from collapsing into premature or excessive certainty about one particular outcome or another.

## Discussion

This qualitative study explored the relationship between prognostic uncertainty and FCR among survivors of OC. To our knowledge it is the first study to focus on bi-directional causal pathways between these phenomena in this population, and to provide evidence of the role of prognostic uncertainty as an effect as well a cause of FCR, an adaptive response as well as an aversive stimulus. Study participants vividly described how uncertainty can not only provoke but ameliorate FCR and sustain hope in the face of an unknown and threatening future. They further reported using various strategies to cope with uncertainty and FCR, and identified several psychological processes that may mediate the relationships between uncertainty, FCR, and hope. These findings are clearly provisional given their qualitative nature; more research will be needed to confirm the causal nature and direction of the relationships identified. In the meantime, however, these findings generate testable hypotheses and provide the basis for a provisional new theoretical model that can guide future empirical research on the relationship between prognostic uncertainty and FCR among cancer survivors.

**FIGURE 1:**
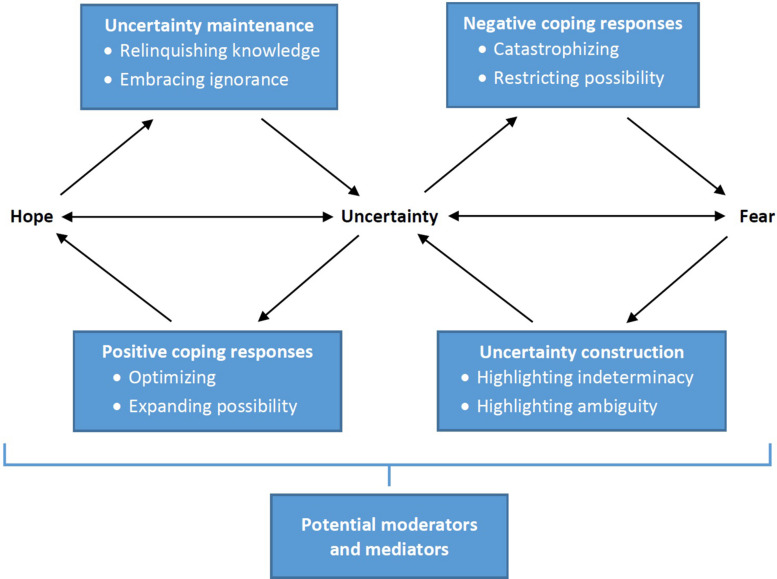
Dual-process equilibrium model of the relationship between prognostic uncertainty, fear of cancer recurrence, and hope.

This provisional theoretical model is presented in Figure 1. Uncertainty occupies the center of this model, affirming both its primary importance in the lives of patients with cancer, and its fundamentally ambiguous, dual nature and function: it is at once a cause and an effect, a source of fear and hope, and a mediating variable between these states. Conventional theories of FCR focus on the direct uncertainty-fear pathway on the right-hand side of the model. For example, the model developed by Fardell et al. (2016) identifies “lack of information” about risk of recurrence as a direct cause of FCR, while integrative models of FCR put forth by Simonelli et al. (2016) and Curran et al. (2017) construe uncertainty as a primary trigger of subsequent cognitive appraisal processes that, in turn, produce FCR. A “blended” model of FCR developed by Lebel et al. (2018) characterizes uncertainty as a primary factor that moderates the effect of internal and external triggers (e.g., physical symptoms) on perceived risk of cancer recurrence, which then leads to FCR. The common feature of all these models is that they situate uncertainty upstream in the causal pathways that lead to FCR, and do not identify uncertainty-specific negative coping processes (e.g., catastrophizing, restricting possibility) that mediate the effects of prognostic uncertainty on FCR. Most importantly, they ignore potential reverse-causal pathways by which fear might induce uncertainty, and the uncertainty construction processes (e.g., highlighting the indeterminacy of prognosis and the ambiguity of prognostic information) that might mediate this pathway.

This new model acknowledges a whole other side of the phenomenon: the critical role of prognostic uncertainty in promoting not only the fear of cancer recurrence but the hope of cancer non-recurrence. The hope-promoting function of uncertainty as a more general phenomenon has been acknowledged in conceptual models put forth by health communication theorists Babrow (1992) and Brashers (2001); however, much remains unknown about the causal pathways connecting uncertainty and hope. In the current model fear and hope are simply inverse responses to the complementary possibilities posed by uncertainty; fear manifests a focus on negative, undesirable possibilities, hope a focus on positive, desirable ones. The model is a dual-process conception that postulates a mirror-image causal pathway by which uncertainty induces hope through the mediating action of positive coping processes (optimizing, expanding possibility), as well as a reverse-causal pathway in which hope induces uncertainty through the mediating action of uncertainty maintenance processes (relinquishing knowledge, embracing ignorance).

This dual-process model thus clarifies that uncertainty is an essential source of both fear and hope, and exactly which of these states predominates at any given time is determined by how individuals balance different positive and negative uncertainty coping strategies. The degree of uncertainty that individuals experience, in turn, is determined by how they balance different uncertainty construction and maintenance strategies. In other words, uncertainty, fear, and hope are interdependent states that exist in dynamic equilibrium and influence one another through different feedback loops. Uncertainty can initiate either a self-perpetuating, vicious cycle of fear, or a similarly self-perpetuating, virtuous cycle of hope. On the one hand, prognostic uncertainty can stimulate primarily negative coping responses that promote FCR, which then stimulates uncertainty construction processes that promote even more uncertainty, which then stimulates further negative coping responses, and so on. On the other hand, prognostic uncertainty can stimulate primarily positive coping responses that promote hope of cancer non-recurrence, which then stimulates uncertainty maintenance processes that promote uncertainty, which then stimulates further positive coping responses, and so on.

More research is needed to elucidate the factors that cause patients to either enter or exit these opposing, self-perpetuating cycles, to experience more or less prognostic uncertainty, to refocus attention on either negative or positive possibilities, or to shift the balance of their psychological responses to uncertainty from fear to hope or vice versa. One important mediating factor may be physicians and physician-patient communication; our data suggest that physicians collude with patients to highlight indeterminacy and ambiguity in prognostic estimates, and thereby co-construct the prognostic uncertainty that both parties need to maintain hope. One important moderating factor may be individual differences in patients’ uncertainty tolerance; past research suggests that uncertainty tolerance influences the extent to which individuals perceive uncertainty and respond to it in negative *vs*. positive ways (Hillen et al., 2017a; Strout et al., 2018; Anderson et al., 2019). These and various other factors may mediate and moderate the causal pathways connecting uncertainty, fear, and hope, and warrant further research.

Our dual-process, equilibrium model of uncertainty provides a guiding framework and set of testable hypotheses for this research. The intermediary processes connecting uncertainty and fear (negative coping responses, uncertainty construction processes) and uncertainty and hope (positive coping responses, uncertainty maintenance processes) can be measured and their effects quantified. In the meantime, our model also raises important normative questions for research on FCR, including how much prognostic uncertainty ought to be constructed and maintained, what negative and positive coping responses are appropriate, and what balance of fear and hope is optimal. Unmitigated fear clearly causes significant suffering and is maladaptive; however, the same may be true for unmitigated hope, which may lead to unrealistic expectations and a “distortion of reality”(Folkman, 2010). More research, both empirical and conceptual, is needed to determine when particular responses are not only psychologically adaptive but morally appropriate, and why.

This study had several limitations that qualify its findings and call for further research. It was conducted at a single institution using a relatively small and racially homogeneous convenience sample of female OC patients with primarily advanced-stage disease. Study recruitment was driven by available study resources rather than thematic saturation; although the repeated occurrence of key themes across the interviews suggested a high degree of thematic saturation, important themes could have been missed. Larger studies, utilizing both qualitative and quantitative methods and more sociodemographically and clinically diverse patient populations, are needed to determine the validity of our findings and theoretical model.

In spite of these limitations, our study provides valuable empirical evidence and a new theoretical model of the relationship between prognostic uncertainty and FCR—and between uncertainty, fear, and hope as more general phenomena. In this model prognostic uncertainty has a dual nature—it is both a cause and an effect, a source of fear as well as hope—and which of these natures predominates at any given time manifests a dynamic equilibrium between dual, opposing processes that promote negative vs. positive coping responses. It remains for future research to confirm our findings, to validate and refine our theoretical model, and to develop interventions that can help patients with OC and other serious illnesses to achieve an optimal balance between uncertainty, fear, and hope.

## Data Availability Statement

The datasets presented in this article are not readily available because they consist of qualitative interview data containing potentially sensitive information, and participants have not given consent for their broader use. Requests to access the datasets should be directed to the corresponding author paul.han09@gmail.com.

## Ethics Statement

The studies involving human participants were reviewed and approved by Maine Medical Center Research Institute IRB. The patients/participants provided their written informed consent to participate in this study.

## Author Contributions

PH contributed to the conceptualization, resources, funding acquisition, supervision, methodology, investigation, formal analysis, writing – original draft, and writing – review and editing. CG contributed to the conceptualization, methodology, investigation, formal analysis, writing – review and editing, and project administration. RH contributed to the formal analysis and writing – review and editing. JL contributed to the conceptualization, resources, project administration, and writing – review and editing. All authors contributed to the article and approved the submitted version.

## Conflict of Interest

The authors declare that the research was conducted in the absence of any commercial or financial relationships that could be construed as a potential conflict of interest.
