# Supplementary material for: Cause or Effect? The Role of Prognostic Uncertainty in the Fear of Cancer Recurrence
Source: Front Psychol. 2021 Jan 15;11:626038. doi: 10.3389/fpsyg.2020.626038 (PMC7843433; doi:10.3389/fpsyg.2020.626038)
Supplement: Supplementary file 1 [file Data_Sheet_1.PDF]

APPENDIX. Interview guide for qualitative interviews

Interview Guide

Understanding ovarian cancer patients' knowledge, attitudes, and experiences regarding prognostic information

---

Approximately 45 minutes

Opening
(*5 minutes*)

This script is intended as a guide. In order to develop rapport, the interviewer seeks to set a conversational tone, diverging at some points from the script. He/she will use discretion in phrasing, use of probes, and additional questions to fully understand the interviewee's perspective and to take into account prior responses. Interview questions may also be modified if results of early interviews suggest that additional questions or probes are needed to fully understand the participants' views on the topics below. All interviews are audio-recorded and transcribed upon completion.

Introduction
Good afternoon/evening. My name is xxxx from the Center for Outcomes Research and Evaluation at Maine Medical Center. First, thank you for taking the time for this interview. As a reminder, the purpose of this project is to learn about your experiences with your ovarian cancer, and how you think about the topic of prognosis, or your future outlook.

- Disclosures
  - Observation
  - Audio taping
  - Measures for safeguarding privacy and confidentiality

- Ground rules
  - No right or wrong answers
  - Some of my questions will be very basic and straightforward, while others may be more complex. Really want to hear your thoughts and opinions.
  - Please be candid, but you do not need to respond to any questions that you are not comfortable answering

- Participant introduction
  - Your first name only

Introduction
(10 minutes)

1. Let's get started. Can you first tell me a little bit about your cancer: how long ago were you diagnosed, what was the stage of your cancer, and what kinds of treatments have you been through (just a brief summary would be fine)?

2. Next, I'd like to know a little about your experiences when you first found out you had ovarian cancer. What were the main questions in your mind when you first heard the news?

3. Thinking back to when your doctor first told you that you had ovarian cancer, did your doctor give you any information about your prognosis?
   By "prognosis" I mean an estimate of what might happen to you, or of what outcomes to expect in the future. Information about prognosis could relate to many different things, including your chances of survival, death, the spread or recurrence of cancer, incontinence or impotence, or other outcomes.

   - What kinds of information did your doctor give you?
   - What future outcomes did he/she talk about with you? *Examples include your chances of survival, death, the spread or recurrence of cancer, incontinence or impotence.*

4. If you were told about your prognosis, how important to you was this information to you, both at the time you were first diagnosed with ovarian cancer, and since that time?

   - Why is information about prognosis important/not important to you?

Experiences with prognostic information and communication
(20 minutes)

Now I'd like to get some more details about how your doctor communicated your prognosis to you.

1. Was anyone with you like a family member or a friend when you learned about your prognosis?

2. How did your doctor introduce the topic?
   - Did you [or anyone you were with] ask for a prognosis?

2. What do you think about how your doctor talked with you about your prognosis?

3. What about in terms of WHAT information he/she gave you? Did you want that information? Did you want other information?

4. Can you tell me about the emotional impact of getting or not getting information about your prognosis. What was it like for you emotionally?

   - At what point in the course of your office visits and treatment did he/she talk about prognosis? At the same time that you received the diagnosis? At a later visit?
   - And what do you think about that timing?

5. How did your doctor communicate information about prognosis to you?

   - Did your doctor talk in terms of risks or probabilities, using numbers? What kinds of numbers did your doctor give you? Percentages [*e.g., "60% chance of x*"]? Proportions [*e.g., "6 out of 10 patients*"]? Expected length of survival [*e.g., "10 years*"]?
   - If your doctor used numbers to talk about prognosis, did he/she give a <u>range</u> of values, rather than a single number—e.g., "50-70%," "12-18 months."
   - Did your doctor use other words to talk about prognosis? *Examples include "high chance" or "low chance," "good" or "bad," "treatable," or "curable".*
     - *What do these words mean to you?*
   - Did your doctor explain where this information came from? *Potential sources include books, journals, computer programs*.
   - Did your doctor use any computer programs or other methods to calculate this information?
     - If so, did he show you these programs directly?

6. What kinds of things did your doctor do to help you understand the information?

   - How much time did your doctor spend giving you this information?
   - Did your doctor use any visual aids, such as graphs or charts?
   - Did your doctor give you any written materials to help explain this information?
   - Did your doctor refer you to any other health professionals to help explain this information? *Examples include nurses, patient navigators, social workers, other physicians.*
   - Did you come away from the visit with a good understanding of the information?
     - If not, what did you not understand?

7. Was there anything that your doctor did that was especially helpful to you with respect to giving you information about your prognosis?

   - Was there anything he/she did that you did NOT find helpful? That you wish had been done differently?

8. Can you tell me more about how you reacted to the information about your prognosis?

   - Did you believe it was true—accurate and trustworthy? If not, why not?
   - Did you believe it applied to your own case and situation? Why or why not?
   - How did you cope with the information then? And how do you cope with your prognosis now?
     - Do you feel hopeful? How do you maintain hope?

9. [If accompanied by friend/family member] How did you feel about having someone [use person's name if given] with you when you learned about your prognosis?

    - How were they involved in the discussion, if at all? Did they ask questions?
    - [if applicable] Did they ask questions you wouldn't have thought to ask?
    - [if applicable] How did you feel about them asking questions?

10. At the time your cancer was diagnosed, did you also search for information about your prognosis from any other sources or people?

    - Which ones/who? (e.g., online sources or websites)
    - Did you find the information helpful? Why or why not?
    - How easy was it to understand the information from the websites?
    - Why did you (or why didn't you) seek out information from other sources?

11. Has information about your prognosis been helpful to you? Why or why not?

    - Did you use this information to make decisions about your treatment? How?
    - Did this information cause worry or anxiety? Hope or reassurance?
    - Did this information cause confusion?

Attitudes and preferences regarding prognostic information and communication
(15 minutes)

1. How often do you think about your prognosis?
    - How does thinking about your prognosis make you feel?
        - If thinking about your prognosis makes you feel badly, how do you cope with that feeling? What sorts of things do you do?

2. How certain or sure do you feel about your prognosis?
    - Why do you say that?
    - How certain do think your doctors are about your prognosis?
    - To what extent do you think it's possible for anyone to know a person's prognosis?

3. We've talked a lot about what your experience was like. Now I'd like to get your thoughts and advice on how to help doctors do a better job communicating with patients about prognosis. In your opinion, how could doctors do a better job talking with patients about their prognosis?
    - Would you want your doctors to give you any information about prognosis?
        - If so, how much information? What kind of information would you want?
        - When and how would you like your doctor to first talk to you about your prognosis?
        - Would you want a friend or family member with you? Who?
        - How often would you want information about your prognosis?
        - What form of information would you want—probe specifically:
            1. Numbers/percentages

2. Qualitative only (e.g., good, bad, worse, better, small chance, large chance, etc)
3. Ranges or confidence intervals
4. Best case/worst case
5. Life expectancy estimates

4. Thank you so much for being willing to talk with me about your experiences. I'd like to ask your advice about one more thing. We are planning to reach out to more women to learn about their experiences. For this study, we asked doctors at MMC to approach women after they completed their first treatment. Do you think that was good timing? Should we have waited longer? Should we have approached women earlier, while they were still in treatment?

Thanks for participating today and providing your opinions. Your comments have been very helpful and insightful.

Your participation is confidential, and your answers will not be attributed in any way to you.

Would you be willing to be contacted again in the future, if we have questions or to see if you're interested in participating in further research studies?

Do you have any questions?
